# A Conspectus of Three Different Forms of Acute Inflammatory Cardiac Disorder

**Published:** 1879-10

**Authors:** Roswell Park

**Affiliations:** Assistant to Chair of Anatomy, Chicago Medical College; Surgeon to South Side Dispensary, etc.; 785 Wabash avenue


### Article II.

A Conspectus of Three Different Forms of Acute Inflammatory Cardiac Disorder. By Roswell Park, A.M., M.D., Assistant to Chair of Anatomy, Chicago Medical College; Surgeon to South Side Dispensary, etc.

An argument often urged against the advantage of fine discrimination in diagnosis, is that in the time and study spent over the signs and symptoms, the actual welfare of the patient may be overlooked. That this is now and then the case, or that we may occasionally meet a successful practitioner who is not an exact diagnostician, few will deny. Nevertheless who, among the cloth, can, now-a-days, afford to disregard anything that will assist towards the most perfect diagnosis possible? And who shall be so callow as to assert that an accurate comprehension of physical conditions is of trifling importance, so that one happens to hit upon the proper drug to exhibit.

The tabular form has always seemed to me the only one in which to set forth what the following conspectus aims to present, viz.: a view, limited but correct as far as it goes, of the diseases under consideration, contrasting each of their features with those of the diseases most resembling them. And if such a table embrace their pathology, their ætiology, etc., so much the more complete it is, and all the more entitled to the term conspectus.

Such the following aims to be. In table I, are presented the three forms of acute inflammatory cardiac disease, not depending on previous cardiac lesions for their causation. It is impossible to give a complete picture of each disease in the column allotted to it, but the effort has been made to so contrast their various features, that under no ordinary circumstances can they be confused.

In table II is presented a refinement of diagnosis made possible by the modern investigations, more especially of the German school of pathologists. Whatever may be said of the visionary character of their work in some directions, it will hardly require an extended experience to recognize the propriety of such a distinction.

It is hoped that the tables will be found complete enough without further explanation here. It only remains for me to mention the writings of Niemeyer, Bauer, Rosenstein and Schrœtter as those chiefly consulted in their compilation; while the various English authors have not been overlooked.

785 Wabash avenue.

### Table I.

*Endocarditis, Myocarditis and Pericarditis Contrasted.*

| Endocarditis. | Myocarditis. | Pericarditis. |
|---|---|---|
| *General considerations.* | *General considerations.* | *General considerations.* |
| In general terms is a parenchymatous inflammation; *i. e.*, without exudation. It is seldom the result of direct irritation, but attacks for the most part those parts most exposed to strain and friction, *e. g.*, the valves. | Its history comprises inflammation, softening, disintegration and accompanying proliferation of the perimysium and consequent formation of cicatrices, or of abscesses, or chronic inflammations of the cardiac tissue. | Is an exudative inflammation, but because this exudate always contains fibrin (in variable quantity) it is not warrantable to attribute it to differences of crasis in the blood. |
| Usually accompanies acute articular rheumatism. Twenty per cent. of cases. | Its ætiology agrees very well with that of endocarditis. When it accompanies rheumatism it is usually an extension of an endo- or pericardial trouble. | Complicates about thirty per cent. of cases of acute articular rheumatism; those especially where several joints are successively attacked. |
| It often accompanies Bright's disease, also acute febrile maladies, puerperal fever, morbilli, etc., the blood of a fever patient acting as an irritant. | In certain cases it may be idiopathic—of independent origin—and have these other cardial inflammations consequent upon it. | It is rarely idiopathic; when it does so occur it is usually when other inflammatory complaints are epidemic, *e. g.*, pleuritis, pneumonia, etc. It may complicate Bright's disease, tuberculosis, chronic endocarditis, or aneurism of the aorta. |
| The existence of a diseased valve may be the cause of a chronic, or very slow, latent endocarditis. | It may be caused by chronic endocarditis, or by typhus, septicæmia, scarlatina, syphilis, etc. | May be the result of infection from septicæmia, puerperal fever, scarlatina maligna, small pox, etc, being a *consequence* and not a complication. |

### TABLE I.—(CONTINUED.)

### *Endocarditis, Myocarditis and Pericarditis Contrasted.*

| ENDOCARDITIS. | MYOCARDITIS. | PERICARDITIS. |
|---|---|---|
| *General considerations.* | *General considerations.* | *General considerations.* |
| It may happen as an extension of myocarditis or pericarditis, or, possibly, of pleuritis or pneumonia. | Thrombi caused by ulcerative or gangrenous disease of the lungs may be dislodged and carried into the coronary arteries, where they set up a circumscribed, purulent inflammation. | May be caused by an extension of myocarditis, endocarditis, pleuritis, pneumonia, etc. |
| | May be the result of a traumatism, though we should have to suppose some predisposition. | May happen as the result of some traumatism. |
| Favorite seat is usually the left side of the heart, except during fœtal life. | Its seat is usually the apex of the left ventricle, or the inter-ventricular septum. | The inflammatory action may be circumscribed or diffused. |
| *Anatomical appearances.* | *Anatomical appearances.* | *Anatomical appearances.* |
| Besides the usual changes in the endocardium, consisting of injection, puffiness, proliferation, fibrinous and chalky deposit, etc., there may be laceration of the endocardium producing aneurism of the heart or chordæ tendineæ, or valves,—adhesions of the same parts or ulcerations. The extension of endocardial inflammation to the inner layers of cardiac muscle, and conse- | Following quickly upon injection and discoloration comes softening with formation of granular detritus and fat globules, and proliferation, cicatrization, etc. If an abscess result there is no removal of products of disintegration nor incapsulation, but extension of the trouble and perforation, with pericarditis if it break externally, or with aneurism or septicæmia, metastasis | The morbid changes may present many modifications,—congestion, extravasation, serous infiltration, proliferation, adhesions, being the possible changes in the pericardium; while the effusion may be insignificant or immense, lightly or heavily loaded with fibrin, giving rise to trifling or serious precipitation upon the heart and pericardial walls; it may be clear or |

23

| | | |
|---|---|---|
| quent loss of tone, may account for subsequent dilatation which so often takes place. | and death as a usual result if it perforate the endocardium. | bloody, very flocculent or even purulent, and very rarely putrid or ichorous. |
| | By making vertical sections the existence of foci may be proved, their favorite sites being the lower anterior or upper posterior surface of left ventricle and left papillary muscle of internal valve. This, of course, in less acute cases.<br>Diminished functional activity may lead to formation of blood clots with subsequent thrombi and vegetations in the cavities of the heart. | The adventitious deposit may be invaded by tubercle; it may be ragged, as often happens when the result of extension of inflammation. It is only in cases of long standing that the substance of the heart suffers material alteration; this consists of serous infiltration and softening, with dilatation, or even with fatty degeneration. |
| *Symptoms.* | *Symptoms.* | *Symptoms.* |
| Supervening upon acute rheumatism it does not always declare itself at the same time with its cause; its appearance may be postponed for weeks. | A mild form seems usually to complicate both endo- and pericarditis. The greater the acceleration and smallness of the pulse, the more probable this view of the case. | Being rarely an independent malady its symptomatology is somewhat ill-defined. |
| In some instances there is pain over the præcordium, and a very frequent but soft, small pulse; these are by no means constant. Palpitation, from laborious and excessive action necessitated by serous infiltration, may be complained of. | When symptoms of cardiac disease appear, either in the course of a rheumatism or of other affections, and physical signs afford negative evidence of either endo- or pericarditis, the diagnosis is more probable. | Palpitation and pain over præcordium are the most usual signs; but excessive pain implies some implication of pleura or lung. |
| Fever may be idiopathic, of inflammatory origin, or it may be of a specific kind when Bright's disease, rheumatism, etc., are present. | Fever only moderate. Temperature but moderately increased; but if metastasis occur, the temperature is suddenly raised. Course rapid, three to eight days. | Fever not at all characteristic; but usually the more fibrinous the exudate, the higher the fever. |

### Table I.—(Continued.)

*Endocarditis, Myocardits and Pericarditis Contrasted.*

| Endocarditis. *Symptoms.* | Myocarditis. *Symptoms.* | Pericarditis. *Symptoms.* |
|---|---|---|
| Pulse may lose its frequency at the outset. If no obstruction to circulation exist, no special dyspnœa is complained of. | Pulse frequent, irregular, unequal. | Embarrassment of circulation and consequently the condition of the pulse, retarded or accelerated, will depend on the amount of effusion. |
| Restlessness, insomnia, delirium and coma, or like symptoms, depend largely on the ætiology. Vide Table II. | In children it may simulate brain disease, being sometimes accompanied by vertigo, heahache, syncope, lethargy or convulsions; these caused by interference with the regular blood supply to the brain. | If the pulse be frequent and small it may resemble some of the fevers of an asthenic type. There may be restlessness, insomnia, coma, etc. Delirium, if present, is of a peculiar type. |
| Dyspnœa and hyperæmia occur, caused also by serous infiltration. | More or less bronchitis, or even œdema of lungs and sanguino-purulent expectoration. Attacks of dyspnœa and præcordial distress may be very severe. | Dyspnœa almost always; especially when caused by compression of the lung, it may be very severe. As if to give most play to the least compressed lung the patient will sit upright or lie on the left side. |
| Rigors, acute swelling of the spleen or pain in its locality, vomiting, hæmaturia and albuminuria, hemiplegia or other signs of emboli or metastasis are of extremely bad import. | If, in addition to the above, signs of metastasis appear, the diagnosis is still more certain. Extreme cyanosis and dropsy, with dilatation, are most unfavorable. Equally so is it when the urine seems to be characteristic of Bright's disease, | It may terminate in chronic pericarditis with relapses, with small, irregular pulse, overloaded veins; or, in dropsy, cyanosis, dyspnœa, and even with possibly fatal œdema of lungs causing slow suffocation. |

| | | |
|---|---|---|
| | In chronic cases there is increased venous pressure, with œdema, hyperæmia, hæmorrhages, dyspnœa, etc., on account of uncompensated valvular disease. | |
| The sequelæ, except when it terminates fatally, are always valvular lesions, and if there have been emboli, any of the numberless lesions that may result from their lodgment in the various organs of the body. | Among the sequelæ may be sudden development of valvular insufficiency, fibroid growths, cardiac aneurism, hypertrophy, dilatation, etc. Death may be caused by collapse, paralysis of the heart, rupture of the heart, œdema of lungs, embolism, etc. | The sequelæ of acute or chronic pericarditis may be: adhesions of heart and pericardium, dilatation, followed by hypertrophy or atrophy, and fatty degeneration from insufficient nutrition, caused by the pressure of the exudation. |
| *Physical Signs.* | *Physical Signs.* | *Pysical Signs.* |
| There is no bulging of the chest. | No bulging of chest. | There may be bulging of the thoracic walls, in young people especially. |
| If cardiac dullness at first be normal, after a few days there may be enough stasis of the pulmonary veins to cause accumulation in the cavities of the heart and some dilatation, with consequent extension of the area of dullness. | Apex beat scarcely perceptible and finally disappearing, pulse small, weak, irregular, feeble, and muffled sounds, constitute the usual physical signs. | The vigor of the heart-beat may be sometimes increased, or if the effusion be copious, it may become imperceptible; sometimes the upright position will make it perceptible. |
| Never any friction fremitus. | Never any fremitus. | Palpation sometimes gives a friction fremitus in the early stages. |
| Whatever changes there may be in the area of cardiac dullness, its limits are comparatively very slightly encroached upon. | The sounds may be clear, but the first indistinct and the second over the aorta very weak; or an unusually loud blowing murmur may be caused by a perforation of the ventricular septum. | Provided the lungs do not intervene there is early an unnatural dullness in front, with resonance behind, varying with the amount of the effusion, the heart seeking the deepest possible position when this |

### TABLE I.—(CONTINUED.)

### *Endocarditis, Myocarditis and Pericarditis Contrasted.*

| ENDOCARDITIS. *Physical Signs.* | MYOCARDITIS. *Physical Signs.* | PERICARDITIS. *Physical Signs.* |
|---|---|---|
| | | is small; when it is large the area forms a triangle with the base downward. It may pass to beyond the left nipple and right border of the sternum. Extension, from day to day of cardiac dullness to the left of the nipple is a sure sign. |
| Substitution of abnormal murmurs at the apex for the systolic sounds, caused by inflammatory change in the parts which produce the normal sounds; according as there is a condition of insufficiency or obstruction, or both, these sounds will differ. | If clots and thrombi occupy any of the cavities, there may be a murmur from friction of the blood upon them. A systolic murmur is often heard at the apex of the heart, but its sounds are quite irregular in strength and succession. | The heart sounds are very feeble or inaudible. The disproportion between extensive dullness and feeble sounds is a diagnostic point. In addition there are usually adventitious friction sounds, which are of longer duration than the normal sounds. Compression may cause dullness of the lower lobe of the left lung, which must not be mistaken for the dullness of pleuritic trouble; pectoral fremitus upon palpation will ensure against this error. |

| *Differential Diagnosis.* | *Differential Diagnosis.* | *Differential Diagnosis.* |
|---|---|---|
| A few days after the onset the area of dullness may be widened, when dilatation of the right heart occurs early. | Must be made largely by a process of exclusion. | The area of dullness begins in the vicinity of the great vessels, and afterwards assumes the triangular form, with the base downward. |
| There will be exaggeration of the heart sounds with the above conditions rather than the reverse. | When, in pericarditis where the effusion is small, the heart's action, previously forcible, becomes weak, we may assume extension of the inflammation to the substance of the heart. When signs of insufficiency in the mitral or aortic valves, or in both, suddenly develop, it may be regarded as more or less characteristic. In this case the murmurs grow weaker and may be lost. Nevertheless, heart murmurs may, of course, disappear from other causes than this. | The apex beat, under the above conditions, becomes more and more imperceptible while the pulse still retains its volume. |
| Although the roughened endocardium is rubbed by the current of the blood, the friction sound is never as distinct. | | The roughened walls give, if any, an almost unmistakably distinct friction sound and often a friction fremitus. |
| This friction sound is isochronic with the heart sounds and may supplant them. | | This friction sound precedes and succeeds the heart sounds, but does not interfere with them. |
| Since the right ventricle lies nearest to and rubs against the thoracic walls, endocardial murmurs — which proceed usually from the left side — are very indistinct over the right side of the heart. | | For the same reason pericardial friction sounds, when the inflammation is diffuse, are heard with equal advantage over either side of the heart. |
| These sounds are transmitted along the blood current, and are not influenced by change in patient's position. | | Endocardial sounds are frequently confined to a small area and are not transmitted; they are liable to change with alteration in patient's position. |

TABLE I.—(CONTINUED.)

*Endocarditis, Myocarditis and Pericarditis Contrasted.*

| ENDOCARDITIS. | MYOCARDITIS. | PERICARDITIS. |
|---|---|---|
| *Differential Diagnosis.* | *Differential Diagnosis.* | *Differential Diagnosis.* |
| The endocardial friction sound can hardly be mistaken for any other. | | The pericardial friction sound is to be distinguished from the pleuritic by its dependence upon or independence of the inspiratory movement. |
| In considering the above signs, the possibility of aneurism, dilatation or hypertrophy or both, old valvular lesions, etc., must be borne in mind. | | In considering the above signs the possibility of aneurism, excessive dilatation of the right auricle, infiltration of the edges of the lung or retraction of its borders, etc., must be borne in mind. |
| *Prognosis.* | *Prognosis.* | *Prognosis.* |
| While life is seldom threatened and recovery usually follows, it is at the expense of some more or less permanent lesion of he circulatory apparatus. Vide Table II. | While there may be a relative cure, the heart remains in no condition to stand sudden or unusual demands. In general terms the prognosis is unfavorable. | The general tendency is usually to recovery in from one to six weeks, according to the severity of the case. In complicated cases the complication is usually of most importance, and the prognosis must be varied accordingly. |

### TABLE II.

### *Varieties of Endocarditis.*

| ULCERATIVE OR DIPHTHERITIC. | VERUCCOSE OR SUBACUTE. |
|---|---|
| Called diphtheritic because of the similarity of its processes with those of diphtheria; so acute and malignant is its nature. | Called "veruccose" because of the appearance of its lesions; termed subacute because it supplies the transition to the chronic, and has many points in common with it. |
| *Pathology.* | |
| The usual locality of the lesions is the left side of the heart; the valve flaps and appendicular and ventricular walls especially. Parenchymatous inflammatory changes and proliferation with subsequent softening, are very rapid, so that ulceration may take place before pus has time to form.<br>Along with these changes are coarse, parenchymatous alterations in various abdominal glandular organs. The spleen is enlarged, even if it contain no emboli. | Usual site of the lesions is the left side of the heart (except when it occurs during fœtal life) and those surfaces of the valves most exposed to friction of the blood current. Inflammatory changes cause a veruccose, organized exudation, of more stable and enduring character. On this the constantly passing blood precipitates fibrin, often in polypoid tufts. |
| Micrococci form a prominent element in the *débris* of softened cardiac tissue. | Micrococci are never found; moreover there is no such *débris* in this form. |
| Embolic infarctions, with consequent abscesses, frequently result, the emboli acting as infective excitants of inflammation; multiple, capillary emboli being a characteristic. | The tufts or threads caused by fibrin deposited by the blood cause larger emboli when swept into the blood current. Their action is mechanical, and not infectious. |
| Complications with myocarditis or pericarditis are frequent; they are often caused by emboli of the coronary vessels. | These complications are frequent, but not from the same cause. |
| Extravasations into the brain meninges occur frequently. | These extravasations occur very rarely. |
| Embolic obstruction of larger vessels of the brain, and metastatic abscesses in the brain, are infrequent. | These lesions are more frequent, because the emboli are larger. |
| Valvular or cardiac aneurisms may be formed in spots weakened by softening and ulceration. | Are never formed. |

TABLE II. — (CONTINUED.)

*Varieties of Endocarditis.*

*Etiology.*

| ULCERATIVE OR DIPHTHERITIC. | VERUCCOSE OR SUBACUTE. |
|---|---|
| Acute rheumatism, especially those cases unaffected by number of joints involved or degree of pain, is a prominent factor. | Acute rheumatism, without reference to its severity or number of joints attacked, is the most prominent factor. |
| It occasionally occurs during puerperal fever, and is often accompanied by undoubted diphtheritic manifestations on the genitalia; *e. g.*, the endometrium. | A recurrent form is frequently developed during pregnancy and the puerperal state. |
| Is an occasional result of pyæmic and septicæmic disease. | Is an occasional result of the acute exanthematous diseases of children. |
| The existence of old endocardial changes, *e. g.*, thickened or retracted tissue, is often favorable ground for the development of ulcerous processes. | Old valvular disease is a frequent factor in the etiology. |
| Age over forty seems comparatively exempt. | No age particularly exempt. |

*Symptoms.*

| TYPHOID FORM. | PYÆMIC FORM. | |
|---|---|---|
| *Marked by more general constitutional disturbance.* | *Character of fever and occurrence of metastases constitute the characteristics.* | |
| If patient has had arthritic pain for some time, it suddenly gives way, while fever still remains high. | Begins with severe chill, with regular or irregular recurrence. | No prodromal symptoms. If it supervene on some other disease, neither the intensity nor type of fever are altered, unless this happen after convalescence has begun, when there may be renewed fever, and patient may complain of palpitation. |
| Or if fever has gone down, a chill comes on, followed by fever and sweating. | Roseolar, petechial or hæmorrhagic spots on skin, or even pustular eruptions attract attention. | Chill or shivering fits with perspiration only when embolism takes place. |

TABLE II. — (CONTINUED.)

*Varieties of Endocarditis.*

| ULCERATIVE OR DIPHTHERITIC. | | VERUCCOSE OR SUBACUTE. |
|---|---|---|
| | *Symptoms.* | |
| TYPHOID FORM. | PYÆMIC FORM. | |
| Defined local complaint, except of palpitation, seldom made. | Same. | Palpitation and shortness of breath may be complained of. |
| Temperature high but variable. Pulse quick, soft, small. Tongue dry. Lips have a sooty coat. | Same group of features obtain. | Fever of intermittent type. Pulse quick but not hard. Tongue and lips present ordinary febrile appearances. |
| Vomiting not infrequent. Diarrhœa and constipation alternate. | Jaundice not infrequent. Diarrhœa with bloody stools. | Disturbances like these are rare. |
| More or less meteorism. | More or less meteorism. | No meteorism. |
| Spleen enlarged. | Spleen enlarged. | Spleen not enlarged, unless plugged by emboli. |
| Delirium and coma gradually supervene. | Same. | Delirium and coma rare, unless caused by emboli. |
| Urine and fæces passed involuntarily. | Dejections involuntarily passed. | Disease rarely reaches such an alarming stage. |
| Urine dark, containing albumen and sometimes blood. | Albuminuria a constant feature. | No albuminuria or hæmaturia, unless from emboli in kidneys. |
| A loud, systolic murmur is heard; occasionally also a diastolic murmur, best heard over the aortic ostium. | Same. If a diastolic murmur is heard, it is because the trouble is mostly confined to the aortic orifice. | A systolic murmur is heard, with maximum intensity, over apex and mitral valves, and with or without the first cardiac sound. Even if the aortic orifice is affected, the systolic murmur usually drowns the diastolic. This murmur is systolic or diastolic, according as insufficiency or stenosis predominate. |

### TABLE II. — (CONTINUED.)

*Varieties of Endocarditis.*

| ULCERATIVE OR DIPHTHERITIC. | | VERUCCOSE OR SUBACUTE. |
|---|---|---|
| | *Symptoms.* | |
| TYPHOID FORM. | PYÆMIC FORM. | |
| Cardiac dullness depends on the situation and area of the lesion. | Same. | There is extended cardiac impulse, but no dullness, unless in the very late stages. |
| *Symptoms common to both forms.* | | |
| Pericardial complications may cause considerable change in the above symptomatology. | | Pericardial complications may more or less disguise the above features. |
| Respiratory disturbances are rendered remarkable by the discrepancy between the dyspnœa and the perceptible pulmonary lesions. They are usually caused by obstruction of the pulmonary vessels. | | While respiratory disturbances are not as marked, usually, they have more or less of this same characteristic discrepancy. |
| The general constitutional disturbance, *e. g.*, *fever*, is far more constant than the cardiac symptoms. | | General constitutional disturbance is never marked, so far as the effects of this form are concerned. |
| Temperature often falls, in a few days, below normal. Character of a remittent fever assumed, though chills may occur at any time. | | Fever preserves more the intermittent type, and chills very seldom, if ever, occur. |
| Aside from disturbances of the sensorium, paralysis is often met with, and is the usual result of coarse lesions, *e. g.*, extravasations, etc. | | Acute hemiplegia, with sudden loss of consciousness, can only occur as result of embolism. |
| | *Differential Diagnosis.* | |
| Is made difficult by paucity of local symptoms. | | The chief difficulty is in distinguishing accidental murmurs from those indicating actual acute disturbance. |
| Even a systolic murmur may be of accidental occurrence. A diastolic is of more importance; but to estimate it properly the possibility of chronic cardiac disease must be excluded. Even the presence of these auscultatory signs is not of so much | | The presence of this form can only be positively diagnosed when the physical signs betray development of valvular disease — temporary or permanent. Having detected a murmur in, *e. g.*, rheumatic cases, it must be proved to be of acute origin. |

### TABLE II. — (CONTINUED.)

### *Varieties of Endocarditis.*

| ULCERATIVE OR DIPHTHERITIC. | VERUCCOSE OR SUBACUTE. |
| --- | --- |
| *Differential Diagnosis.* | |
| import as are changes in their characters. | |
| At first there is a systolic blowing sound confined to area of apex, which then grows weaker there and more audible at the base; later it is complicated with a diastolic blowing sound, and, finally, there is evidence of perfect insufficiency. | The intensification of the second sound in the pulmonary artery, the exact localization of the murmur, and the existence of transverse hypertrophy, even though slight, will all assist in this. Still it must be remembered that a passive dilatation may take place during almost any acute fever. While the sounds of the *right* side should be normal, there may be a sharp accentuation of the second sound of the pulmonary artery, caused by its distension and fullness. |
| Enlarged area of dullness along with the above — except it be owing to pericarditis — confirms the diagnosis, especially when taken with the other and general symptoms. It is distinguished from intermittent by its having no genuine apyretic intervals. | The absence of any hypertrophy or dilatation, or of any obstruction in peripheral arteries, like sclerosis of arterial coats, or of shrinkage of the kidneys, assist in the exclusion of former valvular trouble from consideration. |
| From typhoid, by disproportion in duration of symptoms, their severity, and absence of peculiar temperature curve and abnormal pulse rate. | From myocarditis and pericarditis it may be differentiated by aid of Table I. |
| *Duration.* | |
| When it follows acute rheumatic arthritis, it averages from two to four weeks; when it follows pyæmia or puerperal fever, from three to six days. | Relatively short, because it either leads to chronic valvular trouble, or else terminates fatally through complications with disease of cardiac substance or sac, pleuritis or pneumonia, or through embolism of vital organs. |
| *Prognosis.* | |
| While theoretically recovery is possible, practically no recovery has ever been recorded. | Life is seldom threatened, but absolute recovery is almost impossible. |

